# Case Report: Electroacupuncture combined with transcutaneous auricular vagus nerve stimulation for treating antiseizure medication-resistant juvenile myoclonic epilepsy

**DOI:** 10.3389/fpsyt.2025.1649111

**Published:** 2025-11-11

**Authors:** Yuto Matsuura, Masaaki Murakami, Yuji Kawakubo, Tomomi Sakai

**Affiliations:** 1 Department of Acupuncture and Moxibustion, Tokyo Ariake University of Medical and Health Sciences, Tokyo, Japan; 2 Training in Affiliated Acupuncture and Moxibustion, Acupuncture Center of Tokyo Ariake University of Medical and Health Sciences, Tokyo, Japan; 3 Molecular Psychoneuroimmunology, Institute for Genetic Medicine, Hokkaido University, Hokkaido, Japan; 4 Quantum Immunology Team, Institute for Quantum Life Science, National Institute for Quantum and Radiological Science and Technology (QST), Chiba, Japan; 5 Division of Molecular Neuroimmunology, Department of Homeostatic Regulation, National Institute for Physiological Sciences, National Institutes of Natural Sciences, Aichi, Japan; 6 Institute for Vaccine Research and Development, Hokkaido University, Hokkaido, Japan

**Keywords:** juvenile myoclonic epilepsy, seizure, electroacupuncture, transcutaneous auricular vagus nerve stimulation, neuromodulation

## Abstract

**Background:**

Patients with juvenile myoclonic epilepsy (JME) are frequently resistant to antiseizure medication (ASM) and can have a significantly impaired quality of life (QOL). This case report examines successful treatment of JME using a combination of electroacupuncture and transcutaneous auricular vagus nerve stimulation (taVNS).

**Case presentation:**

A 19-year-old Japanese male with a 5-year history of ASM-resistant JME presented with frequent myoclonic and generalized tonic-clonic seizures, daily premonitory auras, and psychological distress. Despite treatment with sodium valproate and clonazepam, he continued to experience multiple seizures weekly and was unable to attend school due to anxiety. After declining surgical vagus nerve stimulation, he sought acupuncture treatment. Weekly sessions of electroacupuncture (ST36 and LR3) and taVNS targeting the left auricular concha were initiated. From the second session, electroacupuncture was intensified at GV20, GV24, and GB18 due to initial symptom worsening.

**Results:**

Over the course of eight sessions, seizure frequency decreased from multiple daily episodes to a single seizure in the final 4 weeks. Premonitory auras and mild-to-moderate seizures also declined significantly. SF-36 assessments at baseline, 1 month, and 2 months revealed improvements across all subscales except physical functioning, with scores for bodily pain, vitality, and social functioning exceeding national norms at the final assessment. Component summary scores for physical, mental, and role/social functioning also improved consistently. The patient resumed school attendance, experienced reduced anxiety regarding seizures, and reported enhanced social engagement.

**Conclusion:**

This case suggests that combined electroacupuncture and taVNS may be a promising non-pharmacological adjunct in the treatment of ASM-resistant JME, contributing to improved seizure control and multidimensional QOL outcomes.

## 1 Introduction

Epilepsy is a neurological disorder caused by abnormal neuronal discharge in the brain associated with various etiologies (1). The main clinical manifestations include transient disturbances in consciousness, limb twitching, sensory or behavioral abnormalities, and autonomic dysfunction (2). Juvenile myoclonic epilepsy (JME) is the most common out of the four idiopathic generalized epilepsy syndromes in adults, accounting for 5–10% of all epilepsy cases (3). It is one of four idiopathic generalized epilepsy syndromes that occur in adolescents and adults, and is characterized by myoclonic and generalized tonic-clonic seizures (4). Up to 30% of patients with JME are refractory to antiseizure medication (ASM) (5, 6). Moreover, even with adequate seizure control, JME can negatively affect the overall quality of life (QOL) of both adolescents and adults (7). Given these challenges, a comprehensive management approach, including non-pharmacological interventions, is warranted for the treatment of JME.

Neuromodulation is gaining attention as a promising non-pharmacological therapy for psychiatric and neurological disorders (8–10). Acupuncture is a form of neuromodulation that improves central dysfunction by mechanically stimulating peripheral sensitized sites, often referred to as neuroinflammatory spots, and activating peripheral nerves (11). Its efficacy in treating central nervous system disorders has also been reported (12, 13). Recent randomized controlled trials (RCTs) and meta-analyses have shown that acupuncture can reduce seizure frequency and improve QOL in patients with drug-resistant epilepsy. A patient- and assessor-blinded RCT demonstrated that acupuncture administered biweekly for 12 weeks significantly improved seizure control and heart rate variability compared with sham treatment in patients with refractory epilepsy (14). A recent meta-analysis evaluating the adjunctive use of acupuncture alongside Western medicine in epilepsy found that combination therapy enhanced overall treatment efficacy, decreased seizure frequency and electroencephalographic discharges, reduced adverse events, and improved patients’ QOL (15).

Transcutaneous auricular vagus nerve stimulation (taVNS), a technique derived from the principles of auricular acupuncture, is another form of neuromodulation. Recent neuroimaging and clinical studies have positioned it’s as an acupuncture subtype (16). It targets the auricular branch of the vagus nerve, analogous to the auricular acupoints used in traditional acupuncture. Historically, auricular acupuncture has been applied in epilepsy treatment; its antiepileptic effects are hypothesized to result from parasympathetic activation via the auricular branch of the vagus nerve (17). Experimental studies further support this, showing that intradermal auricular electroacupuncture induces state- and frequency-dependent modulation of autonomic function, reinforcing its potential role in seizure control (18). Recent systematic reviews and meta-analyses of RCTs have confirmed the effectiveness of taVNS in reducing seizure frequency and improving QOL, particularly in patients with drug-resistant epilepsy (19, 20).

Preclinical studies indicate that electroacupuncture (EA) and VNS share similar antiepileptic mechanisms, with both demonstrating comparable efficacy in suppressing cortical epileptiform activity in animal models (21). Building on this evidence, combination therapies using EA and taVNS have already been applied in clinical contexts, demonstrating feasibility and potential therapeutic synergy (22). Collectively, these converging lines of evidence provide a strong rationale for exploring EA-taVNS combination therapy in challenging cases such as drug-resistant epilepsy, where conventional pharmacotherapy alone is insufficient. However, only a few studies have specifically addressed the use of acupuncture in JME. Further evaluation of acupuncture’s effectiveness according to epilepsy subtype is necessary to clarify its potential role in clinical practice. Herein, we report on a 19-year-old Japanese man with ASM-resistant JME, involving recurrent seizures and significant QOL impairment, who was successfully treated with a combination of EA and taVNS.

## 2 Case presentation

### 2.1 History of presenting condition

A 19-year-old Japanese man with JME visited our acupuncture clinic with daily myoclonic seizures and occasional generalized tonic-clonic seizures, which had led to functional impairment and reduced QoL. His medical history included bilateral cryptorchidism surgery at 1 year of age, surgery for Legg–Calvé–Perthes disease of the left hip at 6 years of age, hospitalization for cervical spine traction due to cervical vertebral fusion at 9 years of age, and leg lengthening surgery on the left side at 12 years of age. He had no family history of psychiatric disorders, abnormalities at birth or during development, or history of substance use/abuse.

At 14 years of age, the patient experienced a fainting episode and was admitted to hospital where he was initially diagnosed with psychogenic nonepileptic seizures (PNES). Later that year, at a different hospital (a university hospital), he underwent further evaluation, including electroencephalography (EEG), neurological examination, medical interview, and magnetic resonance imaging (MRI), and was subsequently diagnosed with JME by a psychiatrist. Sodium valproate 400 mg/day was initiated at the time, but seizure control did not improve. At the age of 18, in the October, he was treated at another clinic with sustained-release sodium valproate 1,200 mg/day, perampanel hydrate 6 mg/day, and diazepam 5 mg as needed. In November of the same year, his treatment regimen was adjusted to sustained-release sodium valproate 800 mg/day and perampanel hydrate 8 mg/day.

**Figure 1:**
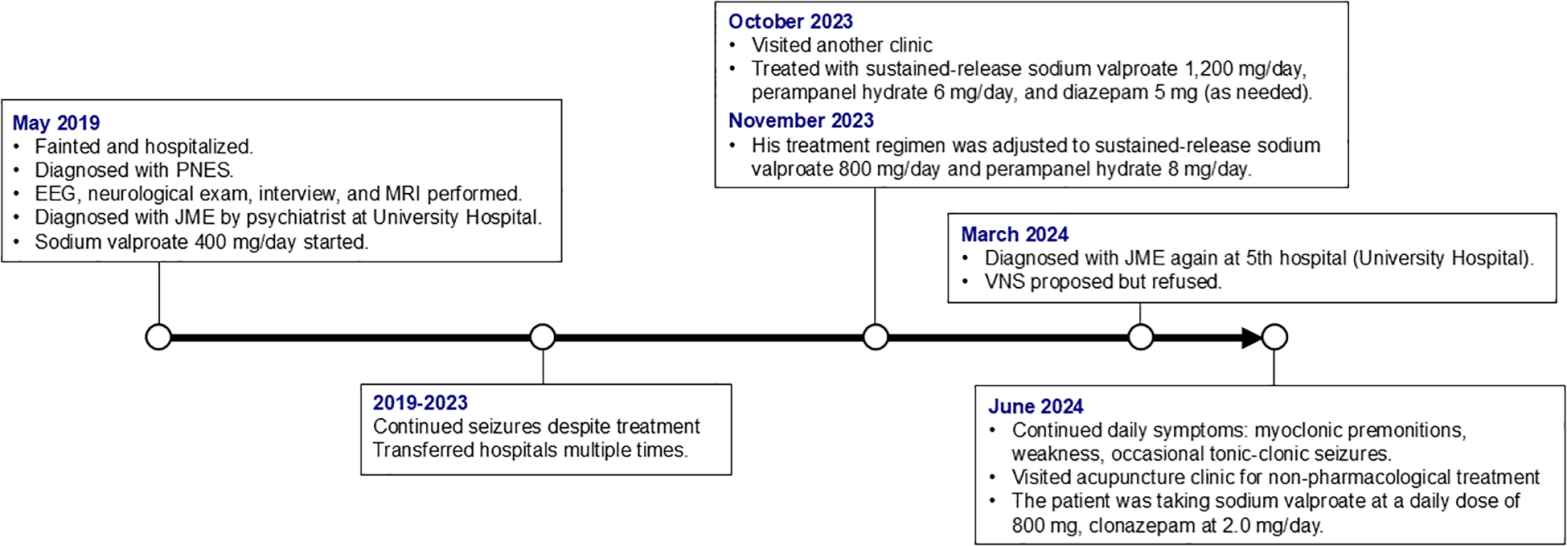
Timeline of key clinical events and treatments. PNES, psychogenic nonepileptic seizures; EEG, electroencephalography; MRI, magnetic resonance imaging; JME, juvenile myoclonic epilepsy; VNS, vagus nerve stimulation.

In the following March, aged 19, he was again diagnosed with JME, at the present university hospital, based on a combination of clinical history, neurological examination, EEG, and MRI (detailed diagnostic results were unavailable). Antiseizure medications at the time included sodium valproate 800 mg/day and clonazepam 1.0 mg/day. In July of the same year, the clonazepam dose was increased to 2.0 mg/day. Because seizures remained poorly controlled, VNS was proposed; however, the patient declined the procedure due to reluctance to undergo invasive surgery. Subsequently, he continued to experience occasional tonic-clonic seizures accompanied by transient loss of consciousness, in addition to premonitions (such as those preceding myoclonic seizures) and weakness almost daily. He visited our acupuncture clinic to improve his symptoms with non-pharmacological treatment (**Figure 1**).

### 2.2 Patient description

At the initial consultation, the patient reported experiencing daily seizure-related symptoms, with progressive worsening in both frequency and severity over the preceding months. The episodes typically began with a premonitory sensation characterized by a feeling of mental cloudiness and an inability to think clearly. Mild seizures (one to three times per day) were characterized by myoclonic jerks in the upper and lower limbs, followed by weakness in all four limbs, leading to collapse, whilst remaining conscious. In addition, generalized tonic-clonic seizures with loss of consciousness occurred approximately once every 1–2 months. Seizures most often occurred at night, particularly after taking antiepileptic medication and before bathing, and each episode lasted approximately 20 minutes.

The patient was a preparatory school student preparing for university entrance examinations. However, due to increasing anxiety about the seizures, he had been unable to attend school for the past month.

At initial visit, the patient’s height and weight were 154 cm and 52 kg, respectively. His blood pressure was 111/54 mmHg, and his pulse rate was 51 beats per minute. Neurological examination revealed normal and symmetrical deep tendon reflexes in the biceps, brachioradialis, and triceps muscles. The patellar reflexes were mildly brisk bilaterally, whereas the Achilles tendon reflexes were normal. Sensory examination did not reveal any abnormalities in the C5–C8 dermatomes. Manual muscle testing revealed normal strength (grade 5) in shoulder flexion, elbow flexion and extension, and finger flexion and opposition in both upper limbs. In the lower limbs, hip flexion, knee extension, ankle dorsiflexion, and plantar flexion were also normal (grade 5), whereas dorsiflexion of the first metatarsophalangeal joints was slightly reduced (grade 4). The trochanteric-epicondylar length was 74 cm on the right and 72 cm on the left. QOL assessment using the Short Form Health Survey (SF-36) yielded the following scores: physical functioning, 57.8; role limitations due to physical health, 15.8; bodily pain, 35.4; general health perceptions, 43.1; vitality, 33.8; social functioning, 11.9; role limitations due to emotional problems, 6.1; and mental health, 27.7. The SF-36 was selected as a validated general health-related QOL instrument that was accessible and feasible to administer in our outpatient setting.

The patient was taking sodium valproate at a daily dose of 800 mg, clonazepam at 2.0 mg/day, a probiotic agent (three tablets/day), and zinc acetate tablets at a daily dose of 50 mg.

### 2.3 Therapeutic intervention

**Figure 2:**
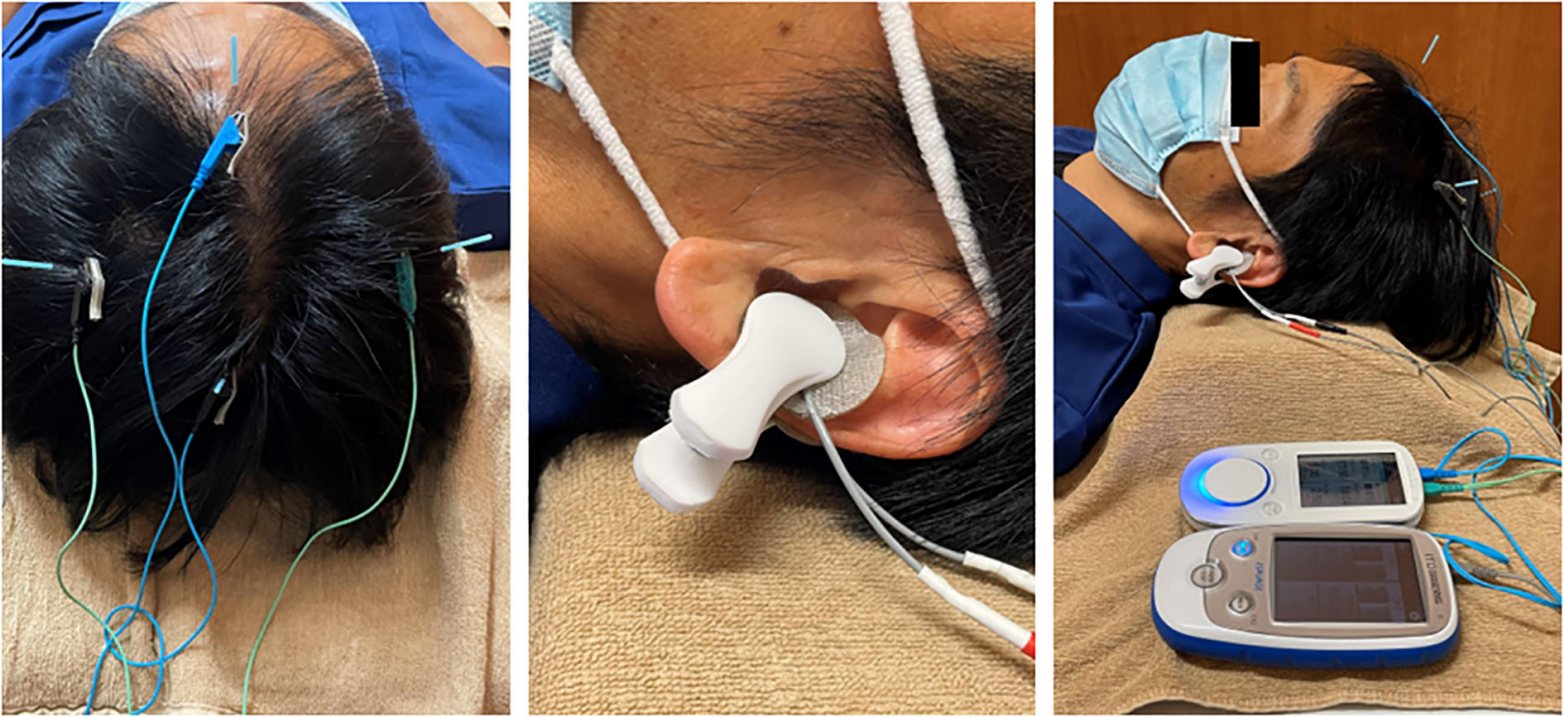
Examples of scalp electroacupuncture and transcutaneous auricular vagus nerve stimulation.

Electroacupuncture combined with taVNS was performed to achieve neuromodulation (**Figure 2**).

This approach has been previously introduced in clinical research (22). In this case, the treatment was administered once weekly by a licensed acupuncturist with 11 years of clinical experience at the Acupuncture Center of Tokyo Ariake University.

Electroacupuncture was performed at ST36 (Zusanli) and LR3 (Taichong) at the patient’s request. Disposable sterile stainless-steel needles (Seirin Co., Ltd., Shizuoka, Japan) measuring 50 mm in length and 0.20 mm in diameter were inserted at bilateral ST36 and LR3. The insertion depth was approximately 40 mm at ST36 and 20 mm at LR3. Electroacupuncture was performed using a low-frequency electrical stimulation device (Picorina^®^; Seirin Co., Ltd.). Electrical stimulation was applied at a frequency of 10 Hz and an intensity of 0.5 mA for 20 minutes. Muscle contractions were not induced during electroacupuncture at ST36. Manual acupuncture with needle retention was performed at GV20 (Baihui) and bilateral HT7 (Shenmen). For GV20, a disposable sterile stainless-steel needle (Seirin Co., Ltd.) measuring 40 mm in length and 0.16 mm in diameter was inserted obliquely to a depth of 20 mm. For HT7, a 15 mm long, 0.16 mm diameter needle was inserted obliquely to a depth of approximately 10 mm. All retained needles were left in place for 20 minutes without manual stimulation. The presence or absence of deqi sensation was not specifically sought.

taVNS was performed using a low-frequency stimulator (ESPURGE^®^, ITO Co., Ltd., Tokyo, Japan). Electrodes were applied to the left auricle and positioned to sandwich the cavum conchae from the anterior and posterior aspects. Electrical stimulation was delivered at a constant intensity of 8 mA, 25 Hz, and pulse width of 250 µs for 20 minutes, adjusted to a level where the patient reported a perceptible sensation without any pain.

### 2.4 Treatment response

#### 2.4.1 Changes in seizure frequency and severity

**Figure 3:**
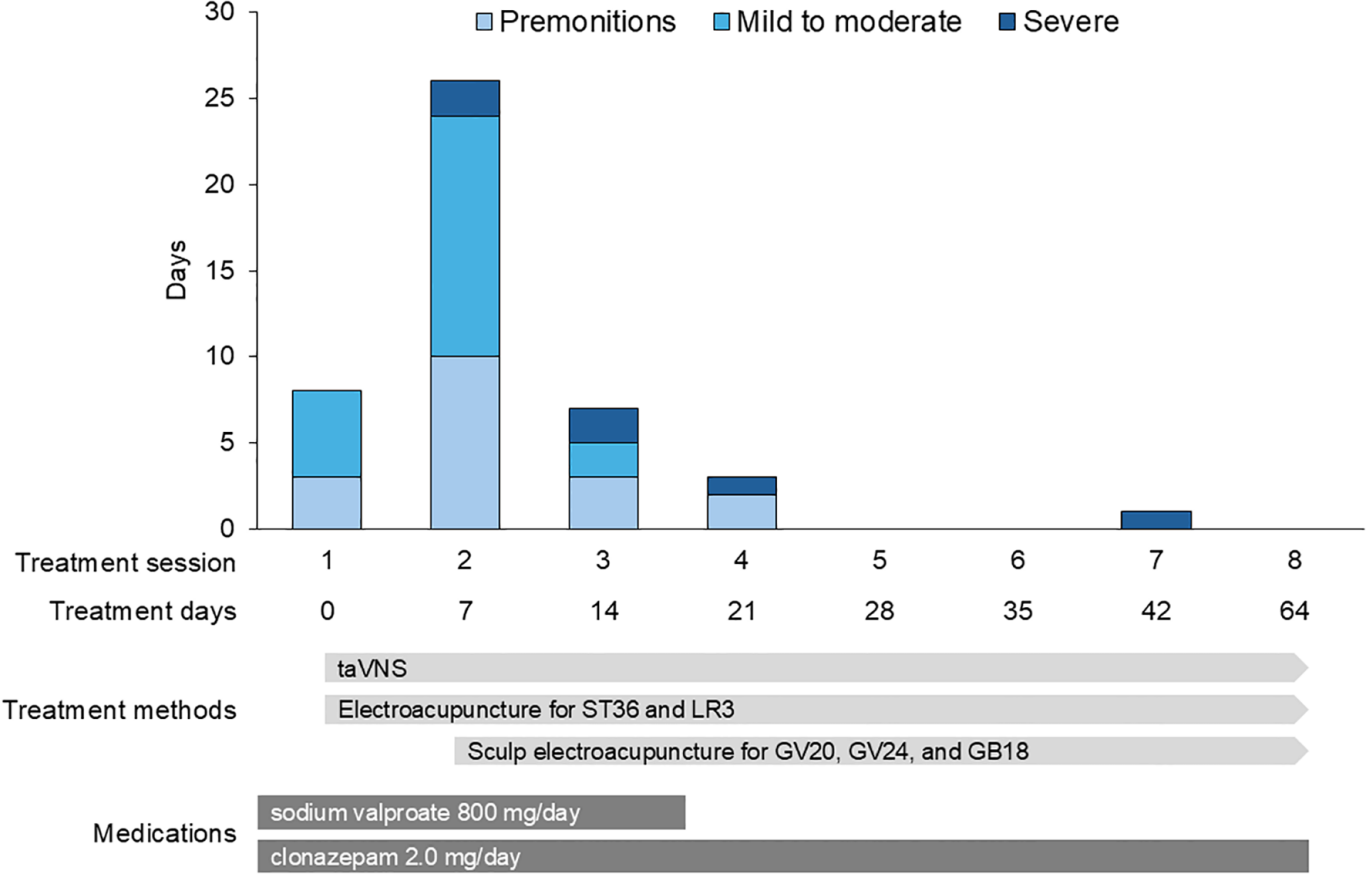
Changes in seizure frequency and severity. The number of premonitory auras, mild-to-moderate seizures, and severe seizures between treatment sessions. There is a marked reduction in seizure frequency and severity following the initiation of intensified electroacupuncture in the second session.

**Figure 3** illustrates the progression of the seizure auras and episodes. Between the first and second sessions, the patient experienced 10 premonitory auras; 14 mild to moderate seizures, primarily occurring once or twice daily after dinner and medication intake; and two severe seizures. One of the severe episodes involved loss of consciousness outdoors, resulting in emergency transport. Due to symptom exacerbation, electroacupuncture was intensified, starting at the second session. The stimulation was performed at GV20 (Baihui), GV24 (Shenting), and bilateral GB18 (Chengling). Disposable stainless-steel needles (60 mm in length, 0.20 mm in diameter) were inserted obliquely to a depth of 20–30 mm, and electrical stimulation was applied alternating between 30 Hz and 100 Hz, with an intensity of 5.0 mA, for 20 minutes.

Between the second and third sessions, the frequency of symptoms decreased to three auras, two mild-to-moderate seizures, and two severe seizures. Between the third and fourth sessions, the frequency further decreased to only two auras and one severe seizure. The patient experienced only one severe seizure from the fourth to the eighth session, indicating a substantial reduction in seizure frequency and severity.

#### 2.4.2 Changes in QOL

**Figure 4:**
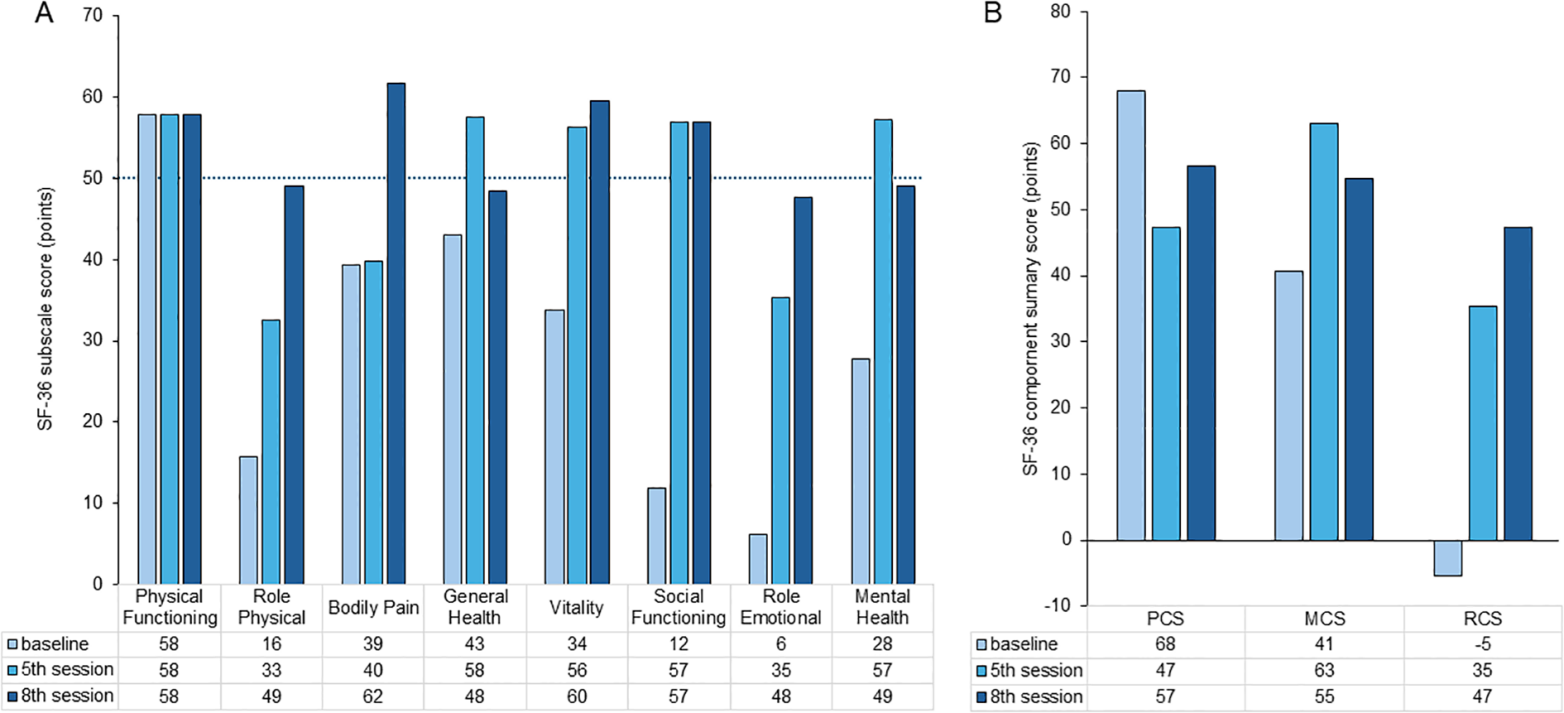
Changes in SF-36 subscale scores **(A)** and component summary scores **(B)**. SF-36 subscale scores measured at baseline, 1 month, and 2 months. Improvements are observable across all subscales, except for physical functioning. Notably, the scores for bodily pain, vitality, and social functioning exceed national normative values at the final assessment. Trajectories of the Physical Component Summary (PCS), Mental Component Summary (MCS), and Role/Social Component Summary (RCS) scores over time. All three component scores show consistent improvement, indicating a global enhancement in quality of life.

QOL was assessed using the SF-36 at baseline, the fifth session (1 month), and the eighth session (2 months). As shown in **Figure 4A**, improvements were observed across all subscales, except physical functioning. Notably, scores for bodily pain, vitality, and social functioning exceeded the national normative values at the final assessment. Furthermore, **Figure 4B** demonstrates a consistent improvement in all three component summary scores: the Physical Component Summary, Mental Component Summary, and Role/Social Component Summary, indicating global enhancement of the patient’s physical, emotional, and social well-being.

#### 2.4.3 Changes in daily life

As seizure frequency decreased, the patient’s psychological anxiety related to seizure recurrence reduced. This allowed him to resume attendance at school, leading to an increased engagement in social activities. His family reported a noticeable reduction in seizure episodes at home, which alleviated their concerns and improved the overall household atmosphere.

## 3 Discussion

This case report highlights the potential utility of combining electroacupuncture and taVNS in the management of ASM-resistant JME. Despite persistent seizures and significant QOL impairment under standard pharmacological treatment, the patient demonstrated substantial improvement in seizure control with the introduction of adjunctive electroacupuncture and taVNS therapy. Additionally, marked improvements were observed in multiple SF-36 subscales, particularly bodily pain, vitality, and social functioning, exceeding national normative values. This case suggests that combining acupuncture-based therapies with conventional epilepsy management may offer benefits to patients with ASM-resistant JME, particularly in terms of improving seizure outcomes and psychosocial QOL.

A major strength of this case report is the detailed documentation of the clinical course and multidimensional outcomes, including seizure frequency and QOL, associated with a novel integrative approach using electroacupuncture and taVNS in a patient with ASM-resistant JME. This case highlights the feasibility and acceptability of these non-pharmacological interventions in a real-world outpatient setting. However, the findings must be interpreted with caution owing to several limitations inherent in single case reports. First, the absence of a control condition limits causal inferences regarding the efficacy of the interventions. The patient’s strong motivation and expectations, as well as the potential placebo effects, cannot be ruled out. This report does not aim to establish a causal relationship between the interventions and clinical improvements, but rather explore the feasibility and potential of combining electroacupuncture and taVNS in managing ASM-resistant JME. Second, the short observation period restricted the assessment of long-term outcomes and the sustainability of the observed improvements. Although the patient reported clear symptomatic and QOL gains over the course of eight treatment sessions, the relatively brief 2-month follow-up precludes firm conclusions regarding the durability of these effects. Longer-term follow-up with post-treatment assessments will be necessary to determine whether these benefits are maintained over time. Third, the contribution of each component (electroacupuncture *vs*. taVNS) to the observed effects remains unclear due to their concurrent application. Because the two modalities were initiated simultaneously, it is not possible to determine whether one had a greater effect or if the observed benefits were synergistic. Future studies employing a controlled or stepwise design are therefore warranted to clarify the individual and combined effects of electroacupuncture and taVNS. Fourth, the use of a non-disease-specific QOL instrument (SF-36) instead of epilepsy-specific tools such as the QOLIE-31 or QOLIE-10 is another limitation. These epilepsy-specific instruments are widely recognized for their relevance in capturing disease-specific QOL impacts. However, due to copyright restrictions and licensing procedures, and considering the practical constraints of a single-case design in a non-hospital acupuncture clinic, we were unable to utilize those tools. Therefore, the SF-36 was chosen as a validated and readily available alternative. Finally, this report lacks objective physiological assessments such as electroencephalographic data, which could have provided additional insight into the neural mechanisms underlying the clinical improvements. This limitation is compounded by the fact that acupuncturists in Japan are not legally permitted to perform diagnostic procedures such as EEG or MRI. Consequently, the clinical information, including diagnosis and treatment history, relied entirely on the patient’s self-reporting. It was therefore difficult to obtain detailed information regarding the initial diagnosis of PNES. Although the diagnosis of JME was consistently made across multiple medical institutions, we did not have direct access to the detailed diagnostic findings.

The clinical improvements observed in this case may be attributed to the neuromodulatory effects of both electroacupuncture and taVNS on the central neural circuits involved in seizure regulation. taVNS has been shown to reduce seizure frequency and improve psychosocial functioning in patients with structural focal epilepsy, potentially by modulation of brainstem-thalamocortical networks via projections from the auricular branch of the vagus nerve to the nucleus tractus solitarius (23). Similarly, acupuncture has been reported to enhance seizure control and QOL when used in conjunction with antiseizure medications, possibly by modulating activity in cortical and subcortical regions such as the thalamus and limbic system, which are implicated in the generation and propagation of generalized seizures (15). Concurrent improvements in seizure activity and QOL, including emotional, social, and cognitive domains, may therefore reflect synergistic neuromodulatory effects mediated by multiple mechanisms. These may include the regulation of brain networks via stimulation at sculp regions such as GV20, GV24, and GB18 (24, 25); activation of the vagal-adrenal axis through electroacupuncture at ST36 (26); and modulation of brainstem-thalamocortical circuits via taVNS (27). Although each of these mechanisms has been independently supported by basic research, their combined application in this case may have contributed to the functional reorganization of neural circuits associated with symptom improvement in JME. Although these studies did not specifically address JME, their findings support the broader applicability of neuromodulatory interventions as adjunctive strategies across epilepsy subtypes.

In addition to the neurophysiological mechanisms discussed above, the potential psychological effects of the interventions should also be considered. Notably, the patient exhibited marked improvements in several SF-36 domains, including vitality, mental health, and social functioning. Both electroacupuncture and taVNS have been associated with antidepressant effects in previous clinical studies (28, 29). Given the well-documented bidirectional relationship between psychological distress and seizure activity in patients with epilepsy, it is plausible that these interventions may have indirectly contributed to seizure reduction through mood enhancement and stress alleviation (30). Although formal psychiatric assessments were not conducted in the present case, the patient’s qualitative self-reporting, together with the observed pattern of QOL improvements, suggest that emotional well-being may have played a mediating role in the therapeutic outcome. Therefore, the clinical improvements observed in this case may reflect not only direct neuromodulatory effects on seizure networks, but also indirect benefits mediated by enhanced mood and psychosocial functioning.

## 4 Conclusion

This case illustrates that the integration of electroacupuncture and taVNS may serve as a viable non-pharmacological adjunct in the management of ASM-resistant JME. Clinicians should consider incorporating such integrative approaches, especially in cases where conventional pharmacotherapy alone is insufficient. Future studies with longer observation periods, involving different epilepsy subtypes, and incorporating objective physiological assessments should be conducted to verify the potential role of this approach in epilepsy management.

## Data Availability

The original contributions presented in the study are included in the article/supplementary material. Further inquiries can be directed to the corresponding author.
